# Excited-State Charge Separation in the Photochemical Mechanism of the Light-Driven Enzyme Protochlorophyllide Oxidoreductase**

**DOI:** 10.1002/anie.201409881

**Published:** 2014-12-08

**Authors:** Derren J Heyes, Samantha J O Hardman, Tobias M Hedison, Robin Hoeven, Greg M Greetham, Michael Towrie, Nigel S Scrutton

**Affiliations:** Manchester Institute of Biotechnology and Photon Science Institute, University of Manchester131 Princess Street, Manchester M1 7DN (UK); Central Laser Facility, Research Complex at Harwell, Science and Technology Facilities CouncilHarwell Oxford, Didcot, OX11 0QX (UK)

**Keywords:** charge transfer, enzyme catalysis, excited states, photochemistry, protochlorophyllide

## Abstract

The unique light-driven enzyme protochlorophyllide oxidoreductase (POR) is an important model system for understanding how light energy can be harnessed to power enzyme reactions. The ultrafast photochemical processes, essential for capturing the excitation energy to drive the subsequent hydride- and proton-transfer chemistry, have so far proven difficult to detect. We have used a combination of time-resolved visible and IR spectroscopy, providing complete temporal resolution over the picosecond–microsecond time range, to propose a new mechanism for the photochemistry. Excited-state interactions between active site residues and a carboxyl group on the Pchlide molecule result in a polarized and highly reactive double bond. This so-called “reactive” intramolecular charge-transfer state creates an electron-deficient site across the double bond to trigger the subsequent nucleophilic attack of NADPH, by the negatively charged hydride from nicotinamide adenine dinucleotide phosphate. This work provides the crucial, missing link between excited-state processes and chemistry in POR. Moreover, it provides important insight into how light energy can be harnessed to drive enzyme catalysis with implications for the design of light-activated chemical and biological catalysts.

A key regulatory step in the chlorophyll biosynthetic pathway is the reduction of the C17-C18 double bond of protochlorophyllide (Pchlide) to form chlorophyllide (Chlide; Figure 1).[1–3] The reaction, which triggers profound changes in plant development that result in the modification and reorganization of the plastid membranes,[1–3] is catalyzed by the light-driven enzyme protochlorophyllide oxidoreductase (POR). As POR is light-activated it is possible to initiate catalysis by using ultrafast laser pulses, thus allowing the chemistry to be monitored on very short timescales, which are not generally accessible for the majority of enzymes that require rapid mixing strategies to derive detailed mechanistic information. Consequently, POR has become an important model system for studying many aspects of enzyme catalysis.[2–9] The reaction catalyzed by POR involves a highly endergonic light-driven hydride transfer from the *pro-S* face of the nicotinamide ring of NADPH to the C17 position of the Pchlide molecule,[5, 6] followed by an exergonic thermally activated proton transfer, most likely from a conserved Tyr residue, to the C18 position of Pchlide[9] (Figure 1). The hydride and proton transfer reactions occur in a sequential mechanism on the microsecond timescale by nuclear tunneling.[6]

**Figure 1:**
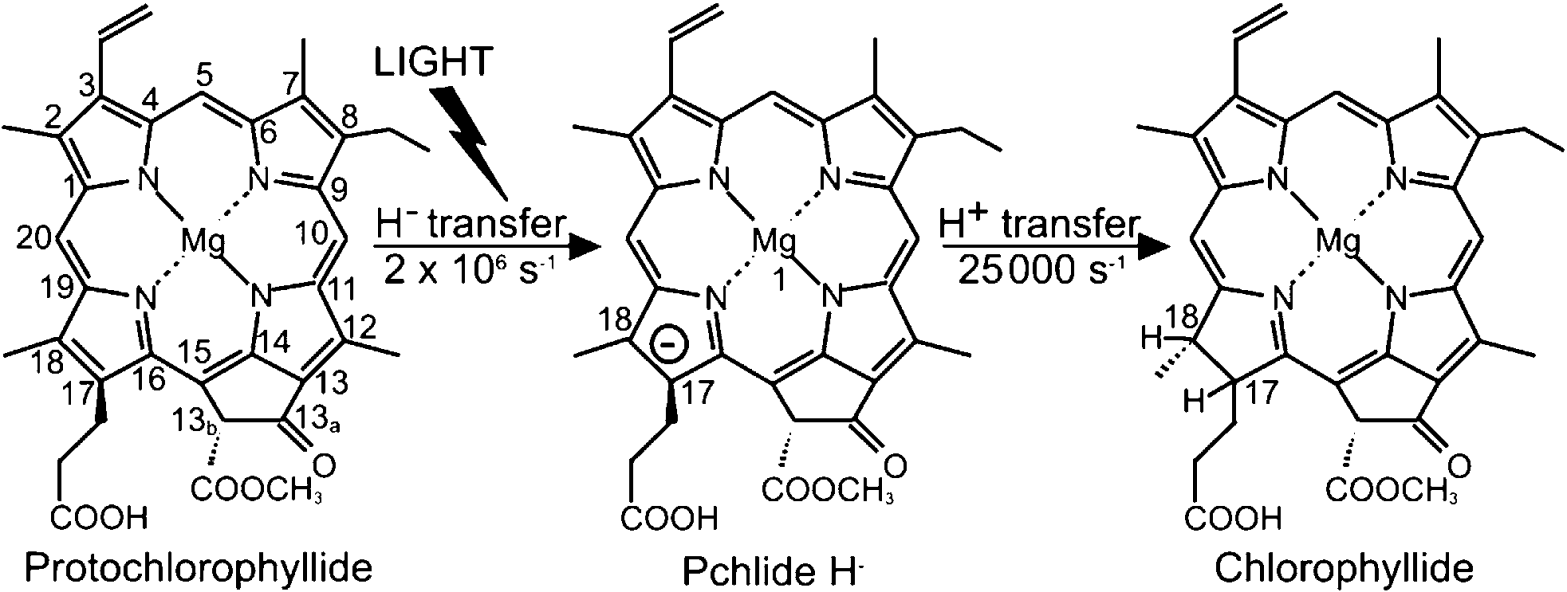
Light-driven reduction of the C17-C18 double bond of protochlorophyllide (Pchlide) to form chlorophyllide (Chlide) is catalyzed by protochlorophyllide oxidoreductase (POR).

Catalysis by POR is also dependent on picosecond excited-state processes within the Pchlide substrate itself.[3] A number of time-resolved transient spectroscopy studies have identified different short-lived Pchlide* species, both in the isolated pigment[11–15] and in the ternary enzyme–substrate complex.[16–20] The Pchlide molecule is intrinsically very reactive with multi-exponential excited-state dynamics[11–16] and the formation of a number of Pchlide excited-state species within a few nanoseconds, including an intramolecular charge-transfer (ICT) complex[11–15] and a triplet state.[13, 15] A detailed understanding of the initial ultrafast steps in the ternary enzyme-substrate (POR-Pchlide-NADPH) complex that lead directly to the chemistry of Pchlide photoreduction has proved more challenging.[16–20] Recent studies have shown that a putative excited-state species with stimulated emission at approximately 675 nm, known as I675*, is not actually a catalytic intermediate in Pchlide photoreduction. Instead, formation of the I675* species is caused by excited-state energy transfer between neighboring pigment molecules in Pchlide-Chlide dimers. This is a result of the accumulation of Chlide product during the course of the measurements and has not permitted a definitive characterization of the excited-state processes associated with POR catalysis.[20] Moreover, conventional ultrafast pump–probe spectroscopy measurements are typically limited to a few nanoseconds, which has prevented the identification of any long-lived excited-state intermediates prior to the hydride and proton transfer chemistry. To overcome this problem we have now used time-resolved UV/Vis and IR spectroscopy techniques that provide complete temporal resolution over the picosecond–microsecond time range. Consequently, this has allowed us to identify all of the excited-state intermediates in the catalytic cycle of POR and to propose a mechanism for harnessing the light energy to drive the subsequent hydride and proton transfer steps on the microsecond timescale.

Time-resolved spectral changes in the visible and infrared (IR) regions after laser excitation at 450 nm have been measured for Pchlide only, the ternary enzyme-substrate (POR-Pchlide-NADPH) complex and for a Y193F mutant ternary enzyme–substrate complex, where the excited-state dynamics were previously shown to be perturbed.[9] Conventional ultrafast pump-visible absorption-probe measurements over timescales up to ≈3 ns were coupled to those using a transient absorption spectrometer that uses an electronically controlled pump-probe delay to provide continuous temporal coverage from picosecond to microsecond. Analogous measurements in the IR region were carried out using the recently developed time-resolved multiple probe spectroscopy (TR^M^PS) technique, where electrical timing again provides nanosecond-to-microsecond delay control of the first probe pulse, combined with the optical delay limits of a few nanoseconds.[21] Global analysis was used to model the time-resolved absorption difference spectra from these measurements by fitting the data to yield species associated difference spectra (SADS).[22] The evolution of the spectral features allows the mechanism of intermediate formation to be determined, whilst the kinetic parameters are indicative of the overall rate of loss of each species, including both conversion to the proceeding state and return to the ground state (see the Supporting Information for further detail). Small differences in fitted lifetimes between the visible and IR datasets can be attributed to differences in solvation (H_2_O in visible or D_2_O in IR) and lower signal-to-noise ratios at longer IR time delays. For clarity only the SADS are shown in the main manuscript, whereas ground-state absorption spectra (Figure S1), raw time-resolved data (Figures S2–8) and an analysis of the global fitting (Figures S9–11) can be found in the Supporting Information.

For the Pchlide only sample in the visible region there is an initial bleaching of the Pchlide ground-state absorption, which appears as a negative band at approximately 635 nm (Figure 2 A and Figure S2). This is accompanied by the appearance of broad excited-state absorption (ESA) bands on either side of the ground state bleach (GSB) band. The initial femtosecond–picosecond dynamics, which represent the S2→S1 transition, vibrational relaxation, and formation of an ICT state, have been described previously[11–15] and are not the main focus of the current study. The subsequent excited-state dynamics could be fitted to three sequentially evolving exponential functions with time constants of 390 ps, 3.2 ns, and 2.7 μs, confirming the model proposed previously.[11–14, 15] The 390 ps component is likely to represent the solvation of the ICT state.[11–14, 15] This is then followed by decay of the S1/ICT excited state into a long-lived triplet state in 3.2 ns, which is characterized by a small apparent blue-shift in the GSB band of approximately 3 nm and the appearance of a new ESA band at approximately 550 nm (Figure 2 A).[11–13, 15] The triplet state then relaxes back to the ground state with a lifetime of around 2.7 μs.

**Figure 2:**
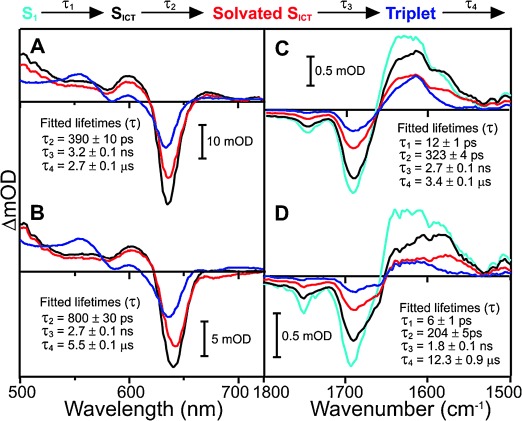
Species associated difference spectra (SADS) resulting from a global analysis of the time-resolved visible data for A) Pchlide only and B) a Y193F-Pchlide-NADPH ternary complex, and the time-resolved IR data for C) Pchlide only and D) a Y193F-Pchlide-NADPH ternary complex after excitation at 450 nm. The data were fitted to the sequential model above the panel as described in the Supporting Information. Kinetic traces showing fits at selected energies are shown in figures S10 and S11.

In the mid-IR region (Figure 2 C and Figure S3) the major spectral negative features at about 1680 and 1750 cm^−1^ represent the C13_a_=O carbonyl frequency and the C=O stretching modes of the carboxyl groups at the C17 and C13_b_ positions, respectively.[15, 22, 23] In the Pchlide only sample the data can be modeled with four component lifetimes. The short-lived S1 component is resolved in the IR data, but not in the visible data, due to the smaller initial step sizes. The lifetimes of the slower components are similar in both the visible and IR measurements. The SADS, which have been fitted with a combination of Gaussian components (Figure S9), show that the proposed solvation of the ICT state (SADS3) is accompanied by changes in the 1550 cm^−1^ region (Figure 2 C and Figure S9). This is in agreement with previous ultrafast time-resolved IR measurements, which suggested that this step is associated with structural changes to the C13 carbonyl and delocalized C=C and C=N modes.[19] This is then converted to SADS4 on the nanosecond timescale, which represents the long-lived triplet state.[15]

In the time-resolved visible data for the Y193F (Figure 2 B and Figure S4) and wild-type POR (Figure 3 A and Figure S5) ternary complex samples the initial GSB is slightly red-shifted to approximately 642 nm, indicating that Pchlide is bound to the enzyme (Figure S1).[4] In addition, the IR difference spectra reveal an extra GSB at 1656 cm^−1^ for the Y193F (Figure 2 D and S6) and wild-type POR (Figure 3 B and S7) ternary complex samples. This is likely to reflect additional hydrogen-bonding interactions between the protein and the bound Pchlide. Also, in contrast to free Pchlide in solution, there is a loss of ESA on the red side of the GSB in the 660–720 nm spectral region, which implies that the relative energy of the ICT state is altered in the ternary enzyme-substrate complexes.[11, 12] Similar findings have also been reported for a pseudo-ternary (i.e. non-productive) POR-Pchlide-NADP^+^ complex where there was a strong blue-shift in the ESA bands associated with the ICT state.[19] Hence, it is possible that hydrogen-bonding effects between residues in the active site of the enzyme and the Pchlide molecule may considerably change or stabilize the ICT state. The negative feature at approximately 675 nm in the POR-Pchlide-NADPH is a result of the Chlide product that accumulates during the course of the measurements.[20] To ensure that this signal did not mask the actual excited-state spectral changes in the POR reaction cycle it was minimized by limiting the length of data acquisition to less than 5 minutes.

**Figure 3:**
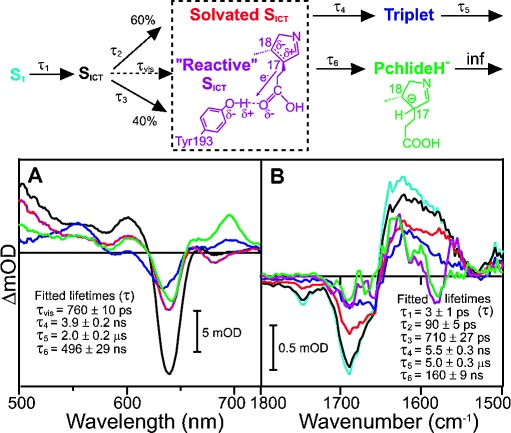
Species associated difference spectra (SADS) resulting from a global analysis of the time-resolved visible data (A) and time-resolved IR data (B) for the wild-type POR-Pchlide-NADPH ternary complex after excitation at 450 nm. The data were fitted as described in the Supporting Information to the branched model above the panel, where 60 % of the ICT state is converted to the solvated ICT state along the “non-catalytic” pathway and 40 % is converted to a “reactive” ICT state along the “catalytic” pathway. Kinetic traces showing fits at selected energies are shown in Figures S10 and S11.

In the wild-type POR ternary enzyme–substrate complex the formation of the hydride transfer intermediate, with a characteristic absorbance band at 696 nm,[4–6] is observed with a lifetime of approximately 500 ns (Figure 3 A), which is similar to that reported previously.[6] In contrast, the same intermediate is not found in the Y193F sample, confirming that photochemistry is impaired in this variant. The other SADS required to model the visible data for wild-type POR or the Y193F variant appear to be very similar to free Pchlide. Hence, from the visible measurements alone it is unclear how the hydride transfer intermediate is formed as it implies a similar mechanism to free Pchlide. On the other hand, the time-resolved IR data for wild-type POR are much more complex than for free Pchlide and the Y193F variant with 2 additional SADS required to accurately model the data (Figure 3 B). The conversion between the S1, S_ICT_, solvated S_ICT_ and triplet states still occurs with very comparable lifetimes and identical peaks in the IR difference spectra to free Pchlide and the Y193F variant (Figure S9). However, in contrast to free Pchlide and the Y193F variant there is an additional “reactive” pathway in wild-type POR from the S_ICT_ state to yield SADS5 and SADS6 (the hydride transfer intermediate). The quantum yield of this pathway was set at 40 % in accordance with previous estimates,[4, 17, 24] and is likely to reflect the orientation of the Pchlide molecule within the enzyme's active site. Hence, based on the time-resolved IR data we propose that there are 2 routes for the excited-state dynamics in wild-type POR-Pchlide-NADPH, which branch after formation of the S_ICT_ state along “catalytic” and “non-catalytic” pathways. This same model can then be applied to accurately fit the time-resolved visible data.

Fitting SADS5 and SADS6 with a sum of Gaussian functions shows several spectral features that are similar to those observed in the “non-catalytic” pathway (Figure 4). For example, the negative features at 1687 and 1656 cm^−1^ correspond to the GSB observed in SADS1-4 (Figure S9). However, there are a number of different structural components in SADS5 and SADS6 (Figure 4) compared to the “non-catalytic” pathway (Figure S9), which allow us to propose a model for the photochemistry along the “catalytic” branch. A down-shift in the peak at 1575 to 1566 cm^−1^ suggests a potential loss of double bond character of the C17-C18 bond.[23] This conclusion is supported by the time-resolved IR data collected for a Chlide only sample (i.e. C17-C18 bond is reduced), which show a significant feature at about 1570 cm^−1^ that is not present in the Pchlide data (Figure S8). The 1575 cm^−1^ downshift is coupled to other excited-state modes at about 1640, 1620, and 1580 cm^−1^ shifting by approximately 12–15 cm^−1^ to higher wavenumbers, suggesting that there is an increase in electron density in other regions of the Pchlide ring system. Moreover, there is a new negative feature at about 1708 cm^−1^, which likely corresponds to loss of signal from the carbonyl stretch of the C17 COOH group.[23]

**Figure 4:**
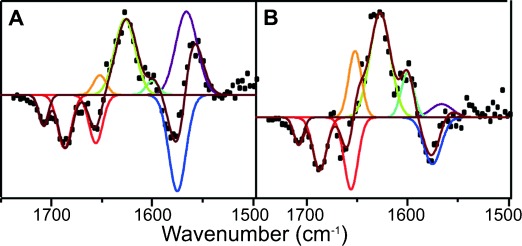
Fitting of SADS5 (A) and SADS6 (B) from the global analysis of the transient IR absorption data for the wild-type POR-Pchlide-NADPH ternary complex to a sum of Gaussian functions. The SADS (black dots) have been fitted with a sum (dark red line) of the following Gaussian functions of fixed position and FWHM (in brackets). Negative peaks: 1708 (11), 1687 (16), 1656 (16) 1575 (20) cm^−1^. Positive peaks: 1652 (15), 1627(24), 1600 (15), 1566 (25).

As the “catalytic” pathway does not occur in the Y193F variant it suggests that the Tyr residue is involved in formation of the “reactive” excited-state species. Taken together, it is likely that excited-state H-bonding interactions between the OH group of the Tyr residue and the carbonyl at the C17 position have an electron-withdrawing effect on the neighboring C17-C18 double bond to create an electron-deficient site (see the reaction scheme in Figure 3). Although vibrational modes associated with the OH group of Tyr are known to be observed at about 1250 cm^−1[25]^ these are beyond the spectral limits of our measurements for direct detection. However, removal of the OH group in the Y193F variant prevents the excited-state H-bonding interactions that create the decrease in local electron density at C17-C18, leading to catalytic inefficiency. Hence, we suggest that along the “catalytic” pathway the ICT state can proceed to a “reactive” ICT state where the C17-C18 double bond becomes highly polarized, thereby facilitating nucleophilic attack of the hydride from NADPH to the C17 position.

In the visible region it is impossible to differentiate between the SADS of the solvated and the “reactive” ICT states, presumably because the changes to the porphyrin ring are only minor. This is expected given that the spectral features in the visible region of the S_ICT_ and solvated S_ICT_ are also identical in Pchlide and Y193F. The dipolar nature of the ICT state in free Pchlide is caused by the presence of the electron-withdrawing carbonyl group on the isocyclic ring of Pchlide,[13, 17, 21] as indicated by the negative peak at about 1743 cm^−1^ (Figure 2, Figure 3 and S9). However, this peak is absent in the “reactive” ICT state, providing further evidence that the electron-deficient site at the C17-C18 double bond is formed as a result of excited-state interactions with the C17 carboxyl group. These data now provide strong mechanistic evidence for how light energy is captured to drive catalysis in POR. Excited-state interactions between the Pchlide molecule and active site residues are required to decrease the local electron density across the C17-C18 double bond, which facilitates the transfer of the negatively charged hydride ion from NADPH. More generally, these findings will provide important, new information on the way in which light energy can be harnessed to drive enzyme-catalyzed reactions. The work identifies a general chemical strategy in which photochemical activation of a light-harvesting substrate (Pchlide) can drive downstream bond breaking/making reactions. This mechanism should have general implications for the design of light-activated chemical and biochemical catalysts.

## Supporting Information

As a service to our authors and readers, this journal provides supporting information supplied by the authors. Such materials are peer reviewed and may be re-organized for online delivery, but are not copy-edited or typeset. Technical support issues arising from supporting information (other than missing files) should be addressed to the authors.

miscellaneous_information
