# Supplementary material for: Excited-State Charge Separation in the Photochemical Mechanism of the Light-Driven Enzyme Protochlorophyllide Oxidoreductase
Source: Angew Chem Int Ed Engl. 2014 Dec 8;54(5):1512–5. doi: 10.1002/anie.201409881 (PMC4531822; doi:10.1002/anie.201409881)
Supplement: Supplementary file 1 — miscellaneous_information [file anie0054-1512-sd1.pdf]

Angewandte Chemie
Eine Zeitschrift der Gesellschaft Deutscher Chemiker

Supporting Information

© Wiley-VCH 2015

69451 Weinheim, Germany

Excited-State Charge Separation in the Photochemical Mechanism of the Light-Driven Enzyme Protochlorophyllide Oxidoreductase**

Derren J. Heyes, Samantha J. O. Hardman, Tobias M. Hedison, Robin Hoeven, Greg M. Greetham, Michael Towrie, and Nigel S. Scrutton**

anie_201409881_sm_miscellaneous_information.pdf

Supporting Information

Experimental Section

Sample Preparation

Wild-type (1) and Y193F (2) POR from *Thermosynechococcus elongatus* were overexpressed in *Escherichia coli* and purified as described (1, 2). Pchlide was purified as described previously (1). Chlide was synthesized by the insertion of magnesium into pheophorbide *a* as described previously (3). For the time-resolved visible spectroscopy measurements samples contained 200 µM Pchlide in the presence and absence of 500 µM POR and 2 mM NADPH in activity buffer (50 mM Tris pH 7.5, 100 mM NaCl, 1 % Triton X-100, 0.1 % 2-mercaptoethanol). For the time-resolved IR spectroscopy measurements samples contained 350 µM Pchlide in the presence and absence of 500 µM POR and 2.5 mM NADPH in D_20 activity buffer (50 mM Tris pD 7.5, 100 mM NaCl, 1 % Triton X-100, 0.1 % 2-mercaptoethanol). Absorbance spectra were recorded using a Cary 50 UV/visible spectrophotometer (Agilent Technologies).

Visible Time-Resolved Spectroscopy

A Ti:sapphire amplifier (hybrid Coherent Legend Elite-F-HE) was pumped by a Q-switched Nd:YLF laser (Positive light evolution-30) and seeded by a Ti:sapphire laser (Spectra Physics Mai Tai). The amplifier output (1 kHz repetition rate, 800 nm centre wavelength, ~120 fs pulse duration) was split to generate the pump and probe beams. A non-collinear optical parametric amplifier (light conversion TOPAS white) was used to generate the pump beam centered at 450 nm, with FWHM of ca. 10 nm. A broad band ultrafast pump-probe transient absorbance spectrometer 'Helios' (Ultrafast systems LLC) was used to collect data (randomly) from ~5 ps to 3 ns with a time resolution of around 0.2 ps. The probe beam consisted of a white light continuum generated in a sapphire crystal, absorbance changes were monitored between 500 and 750 nm. Data from this set-up are referred to as the 'fast' data. A broad band sub-nanosecond pump-probe transient absorbance spectrometer 'Eos' (Ultrafast systems LLC) was used to collect data (randomly) up to 2 microseconds. A 2 kHz white-light continuum fibre laser was used to generate the probe pulses. The delay between pump and probe was managed electronically. For both sets of measurements samples were excited at 450 nm with 0.5 µJ power and a beam diameter of ~ 200 µm. Data from this set-up are referred to as the 'fast' data.

Samples were flowed at a rate of approximately 20 ml/min through a 0.2 mm pathlength quartz cell to ensure that a different area of the sample is excited with each pump laser pulse. After correcting the 'fast' data for spectral chirp the 'fast' and 'slow' datasets were combined by scaling the full

‘slow’ dataset by a fixed factor to match the intensity of ground state bleach feature in the ‘fast’ dataset at similar time points (datasets overlap between ~0.5 to 3 ns).

Infra-Red Time-Resolved Spectroscopy

These experiments were carried out at the Ultra facility (CLF, STFC Rutherford Appleton Laboratory, UK), using the recently developed time-resolved multiple probe spectroscopy (TR^{M}PS) technique (4). Samples in D_2O buffer were contained between two CaF_2 windows, separated by a teflon spacer to give a pathlength of approximately 100 µm. The sample was flowed through the cell and the sample holder rastered to avoid sample damage. For all samples an excitation wavelength of 450 nm was used with 0.5 µJ pulse power and a beam diameter of ~ 150 µm set at the magic angle with respect to the IR probe beam. Difference spectra were generated relative to the ground state in the spectral window 1500-1800 cm^{-1} at time delays ranging between 500 fs and 2 µs. Data were collected for approximately 10 mins per dataset, the spectral resolution was ~3 cm^{-1} and pixel to wavenumber calibration was performed as described previously (5).

Global Analysis

The datasets were analyzed globally using the open-source software Glotaran (6). This procedure reduces the matrix of change in absorbance as a function of time and wavelength, to a model of one or more exponentially decaying time components, as described in the main manuscript, each with a corresponding difference spectrum (species associated difference spectra (SADS)). Errors quoted with the lifetime values are the standard errors calculated during the global analysis.

Previous studies on Pchlide systems have concluded that the fluorescence quantum yield is on the order of 6-9% (7). The overall triplet yield (compared to the entire excited state population) has been found to be around 30% (8, 9). Thus from the total initial excited state population it can be assumed that around 65% returns to the ground state *via* non-radiative processes. Rather than try to exactly fit these diverse processes, which would result in a model with so many parameters that almost anything would have fitted, we have chosen to use a simplified model in which the that the lifetimes quoted for the conversion between states also include contributions from the rates of ground state recovery through both radiative and non-radiative processes. Differences in experimental conditions (e.g. H_2O *vs*. D_2O) could be expected to affect the rates of non-radiative relaxation, hence the apparent disparities in some of the rate constants described here. The purpose of the global analysis is to distinguish the evolution of spectral features in order to determine the mechanism of intermediate formation rather than precise kinetics. The model used to fit the ternary POR-Pchlide-NADPH complex was settled upon after extensive testing. Starting with the simple sequential scheme derived from the Pchlide only data, branches of varying length (1,2, or 3 components) to the ‘A696’ intermediate were tried from each of the S1, S_{ICT}, Solvated S_{ICT} and T

states. The final model was the only one which produced a set of physically sensible SADS and reasonable interconversion lifetimes.

Supplementary Figures

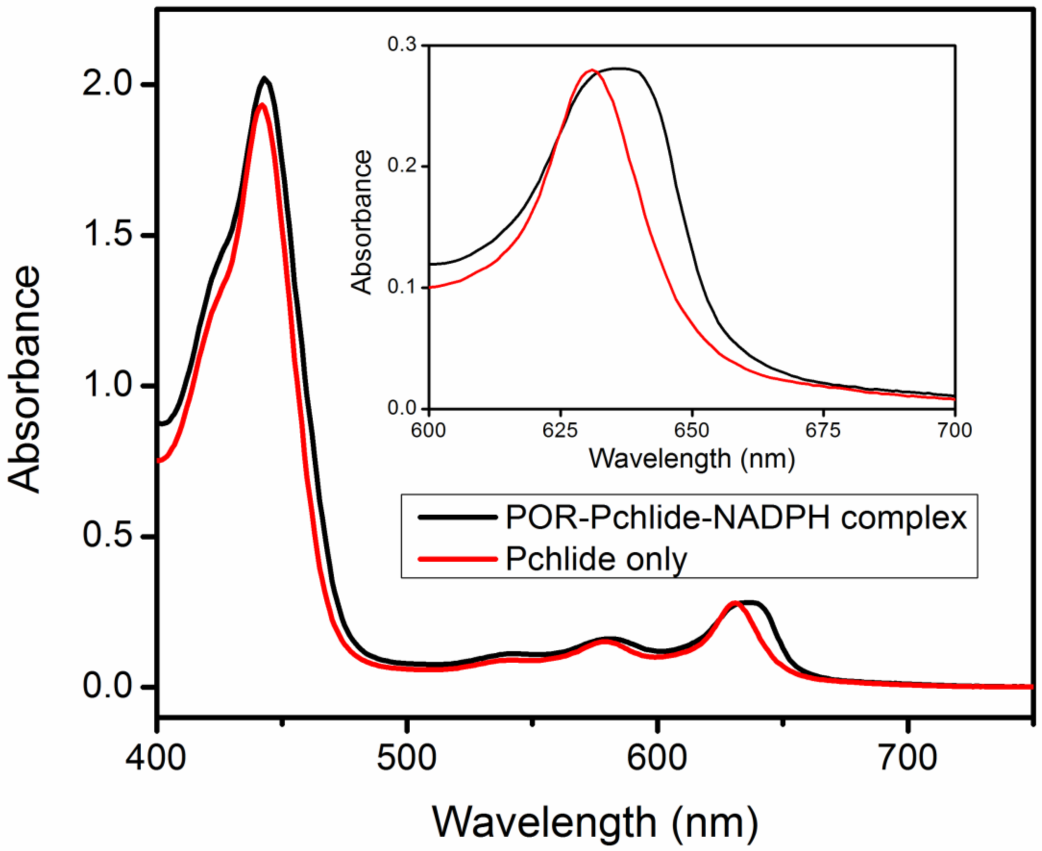

Figure S1. Ground state absorbance spectra of Pchlide only and POR-Pchlide-NAPDH ternary complex samples.

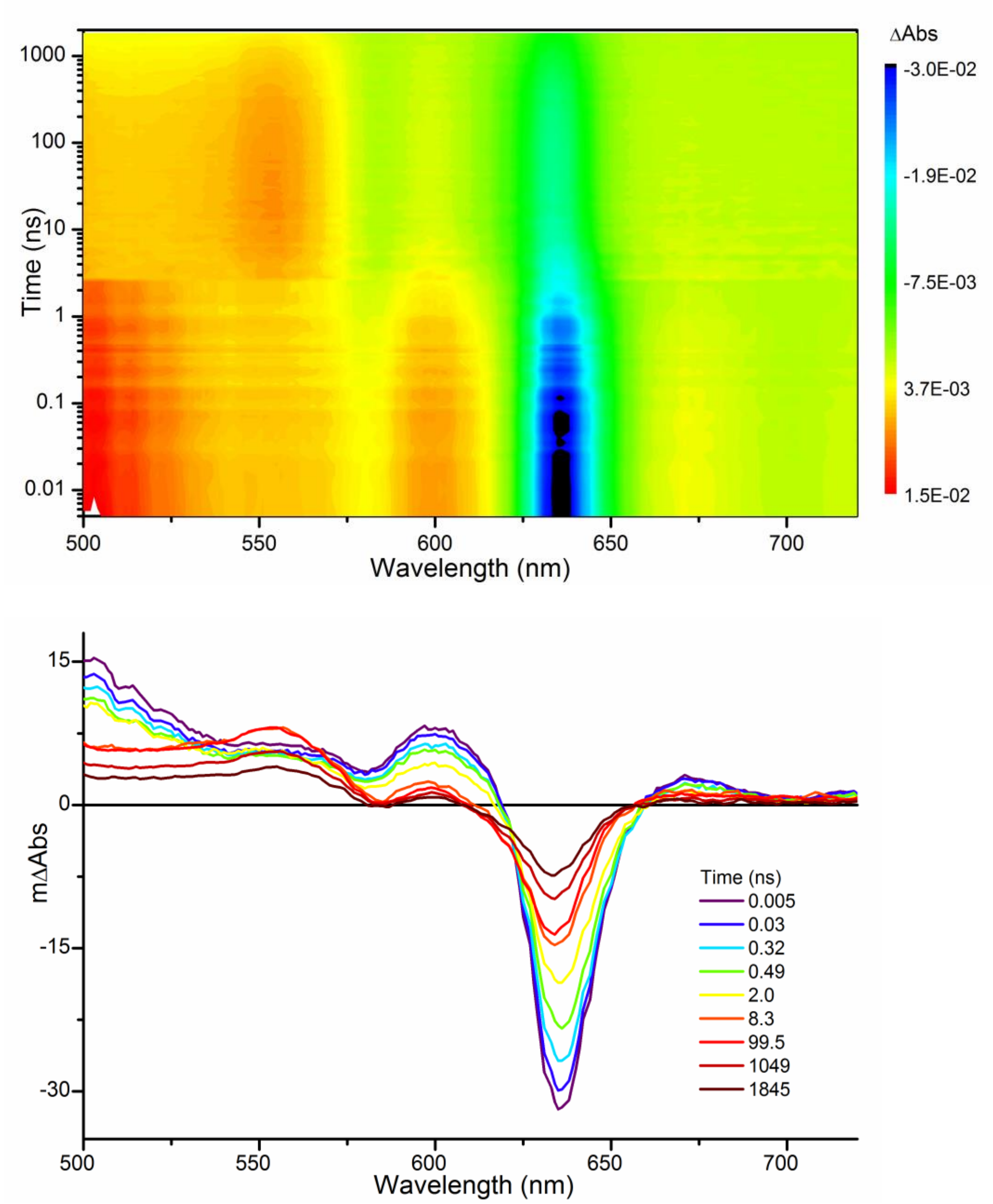

Figure S2. Time-resolved visible spectroscopy data for Pchlide only after photoexcitation with a laser pulse centred at ~450 nm. Time-resolved difference spectra were recorded between 5 ps and 2 μs as described in the Experimental section.

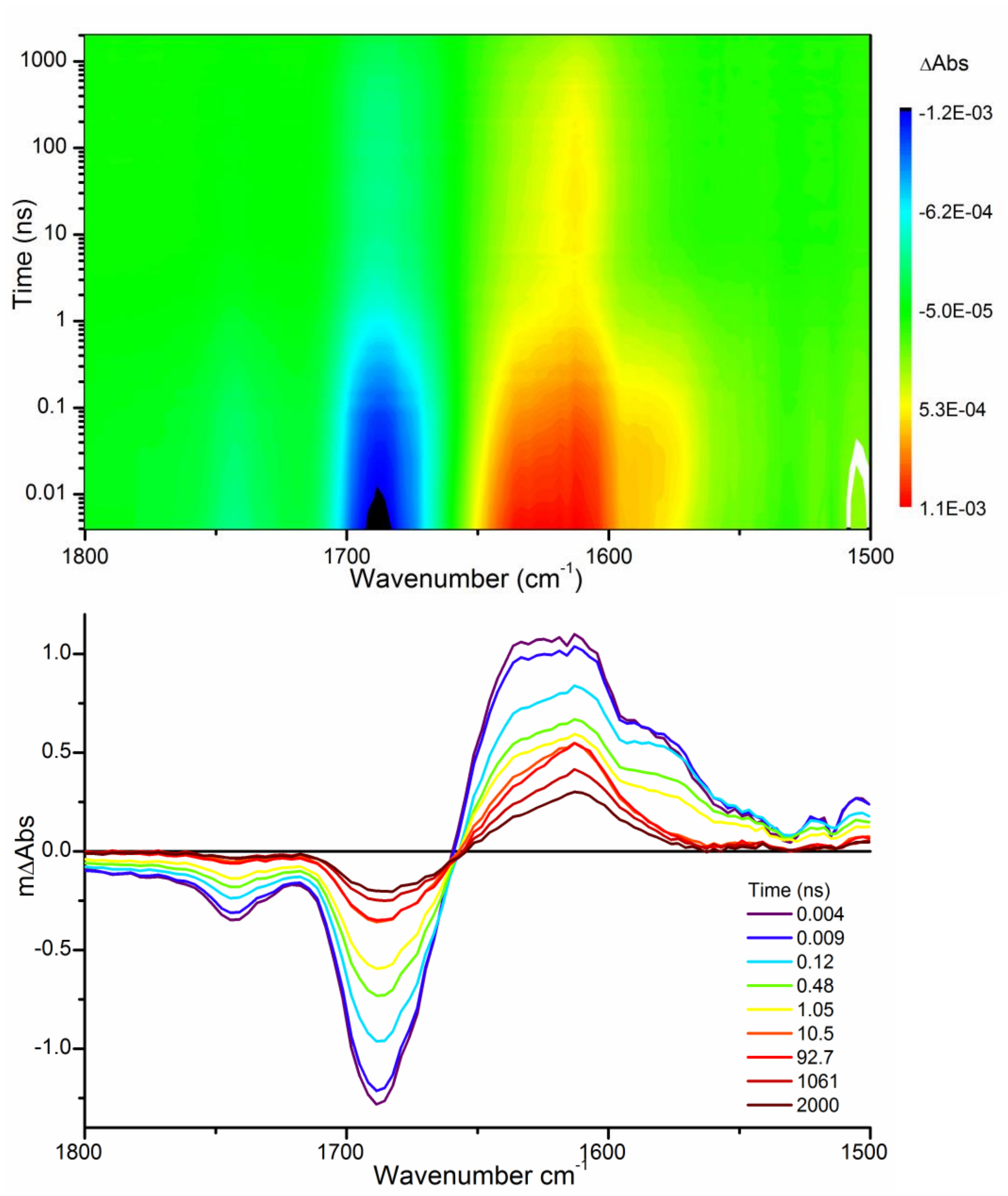

Figure S3. Time-resolved IR spectroscopy data for Pchlide only after photoexcitation with a laser pulse centred at ~450 nm. Time-resolved difference spectra were recorded between 4 ps and 2 μs as described in the Experimental section.

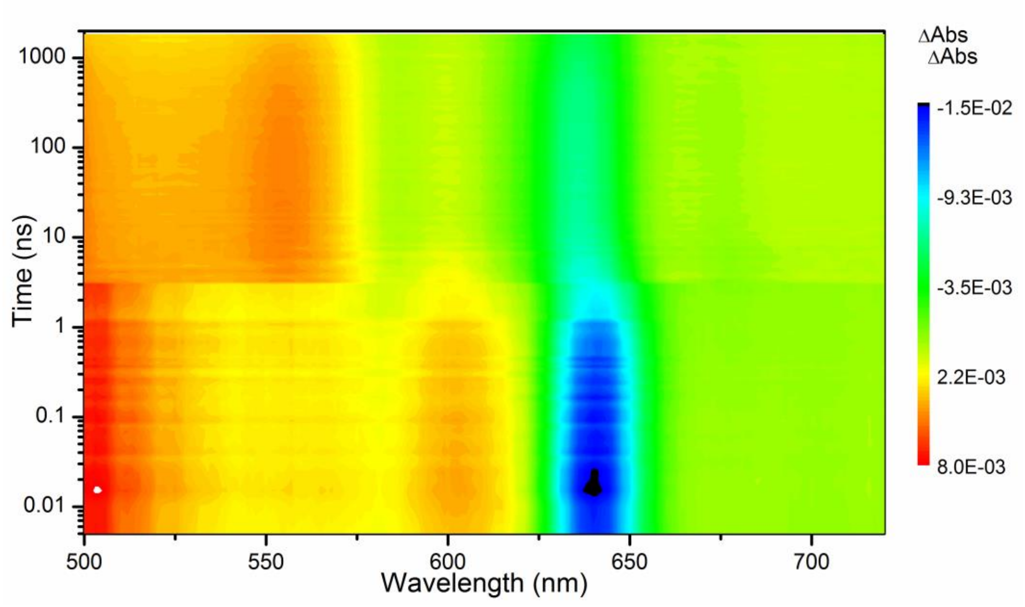

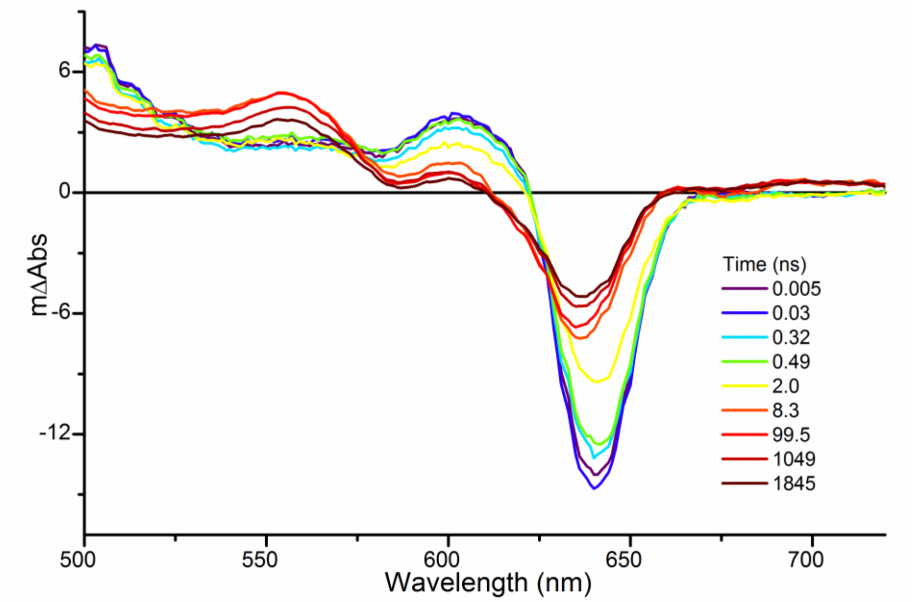

Figure S4. Time-resolved visible spectroscopy data for a Y193F POR-Pchlide-NADPH ternary complex after photoexcitation with a laser pulse centred at ~450 nm. Time-resolved difference spectra were recorded between 5 ps and 2 μs as described in the Experimental section.

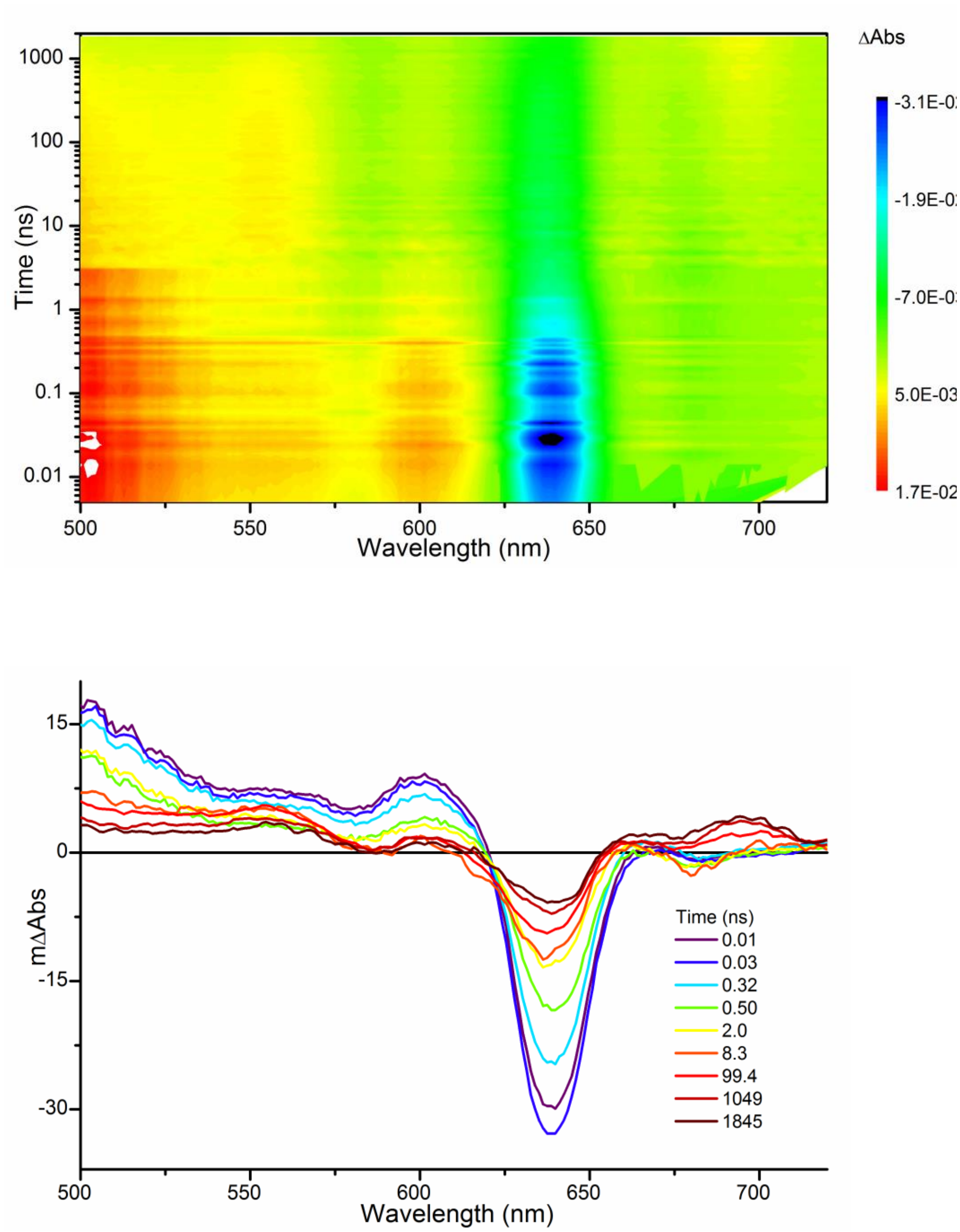

Figure S5. Time-resolved visible spectroscopy data for a wild-type POR-Pchlide-NADPH ternary complex after photoexcitation with a laser pulse centred at ~450 nm. Time-resolved difference spectra were recorded between 14 ps and 2 μs as described in the Experimental section.

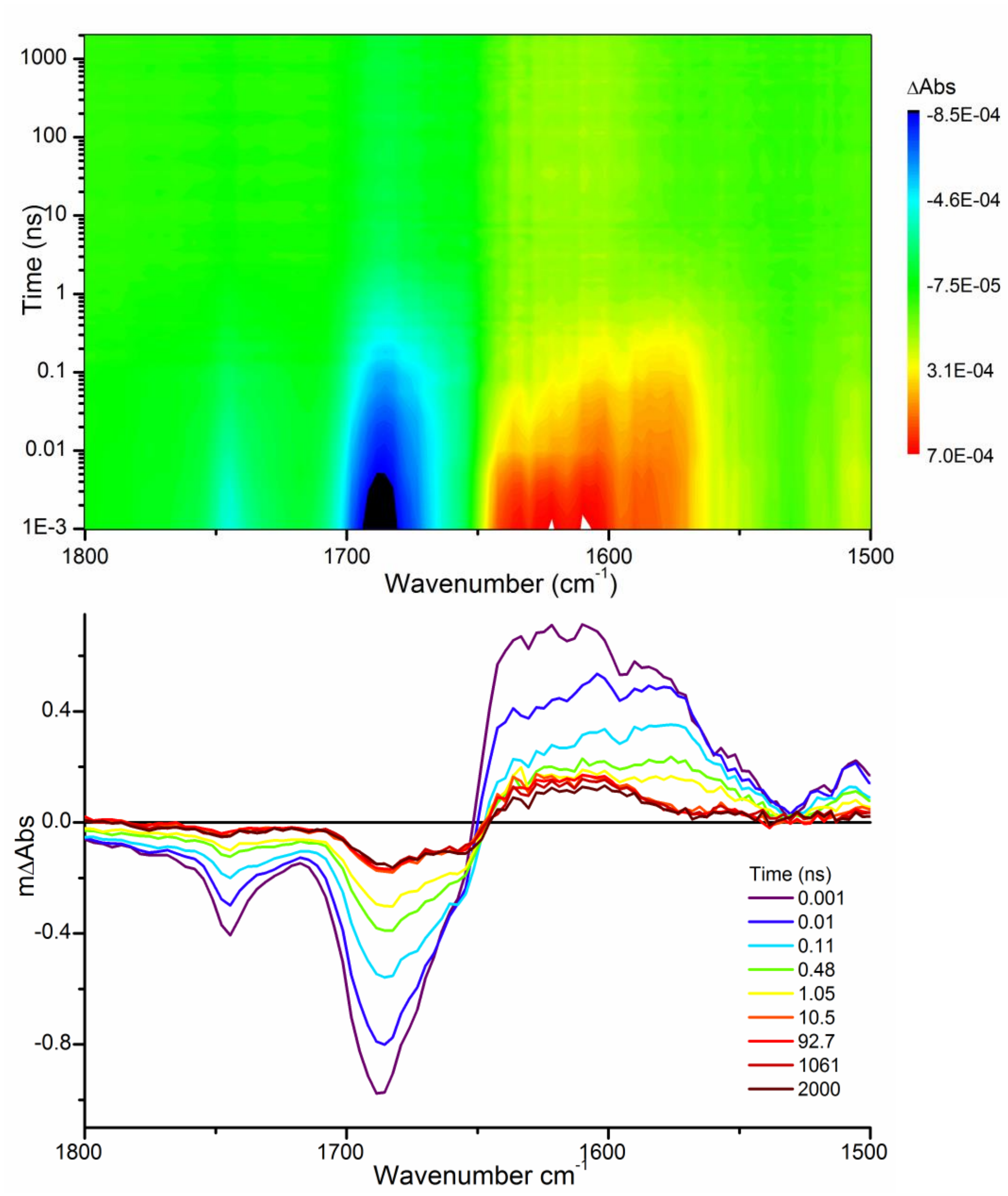

Figure S6. Time-resolved IR spectroscopy data for a Y193F POR-Pchlide-NADPH ternary complex after photoexcitation with a laser pulse centred at ~450 nm. Time-resolved difference spectra were recorded between 1 ps and 2 μs as described in the Experimental section.

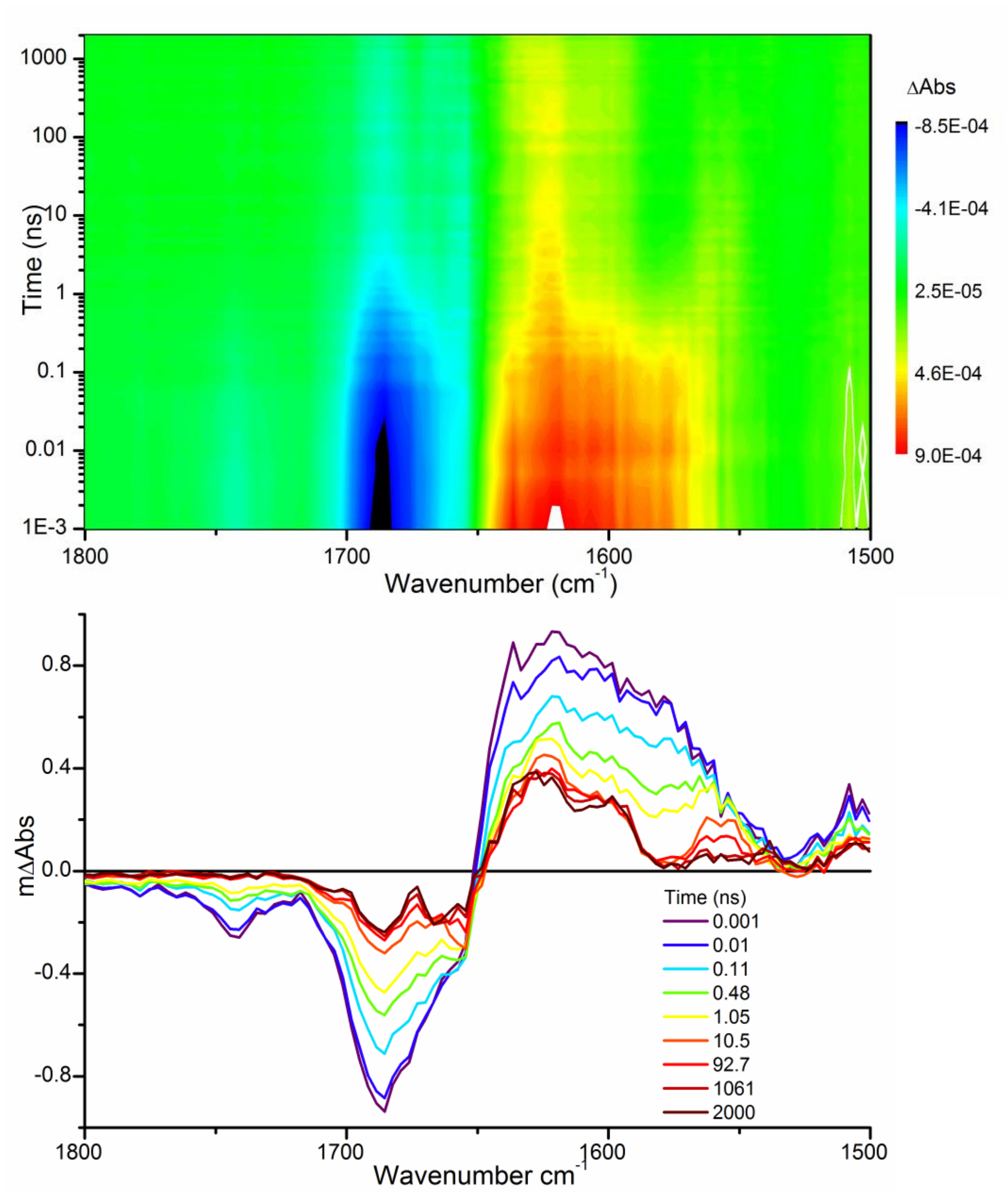

Figure S7. Time-resolved IR spectroscopy data for a wild-type POR-Pchlide-NADPH ternary complex after photoexcitation with a laser pulse centred at ~450 nm. Time-resolved difference spectra were recorded between 1 ps and 2 μs as described in the Experimental section.

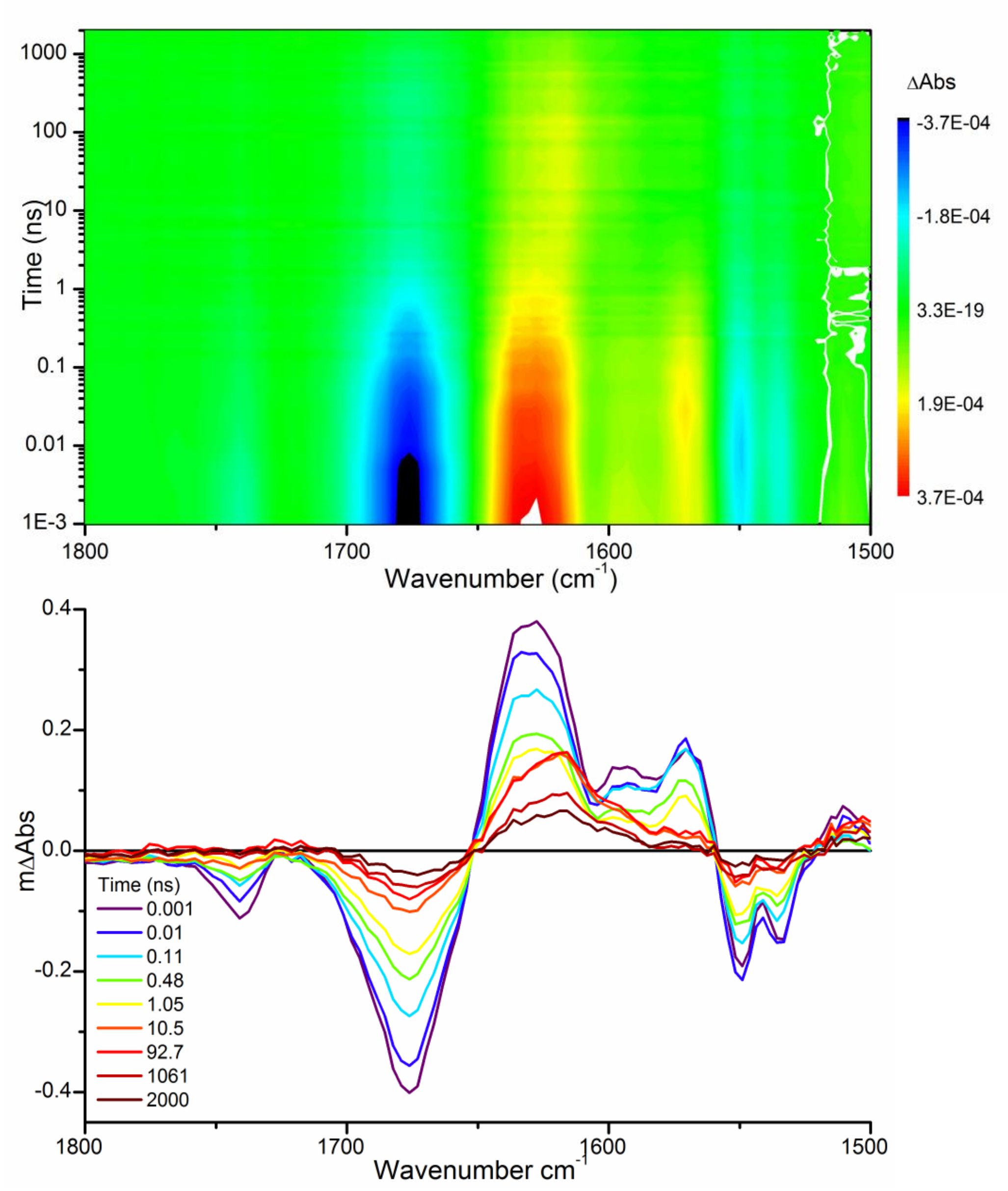

Figure S8. Time-resolved IR spectroscopy data for Chlide only after photoexcitation with a laser pulse centred at ~450 nm. Time-resolved difference spectra were recorded between 1 ps and 2 µs as described in the Experimental section.

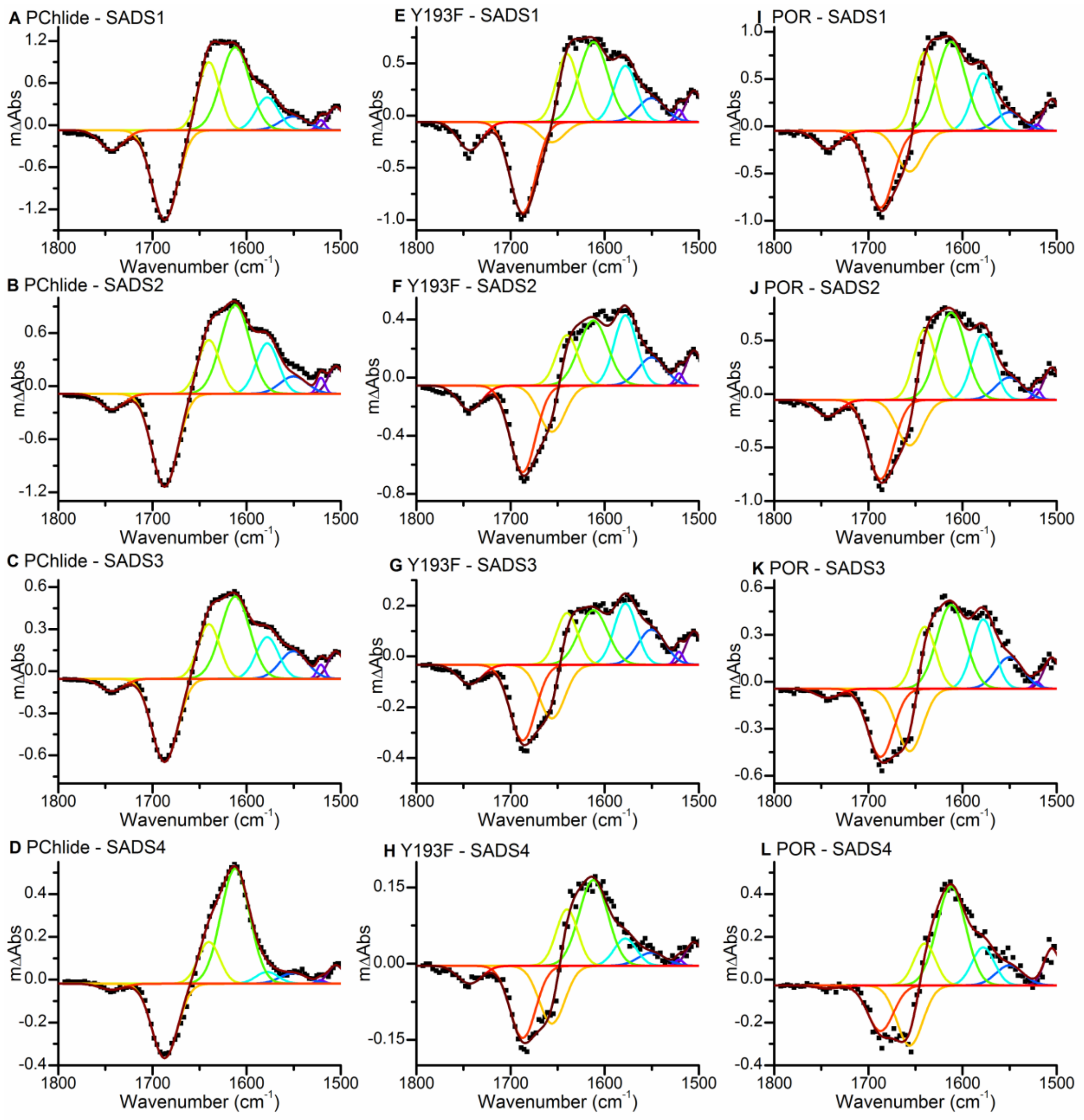

Figure S9. Gaussian fitting of SADS1-4 resulting from the global analysis of the transient IR absorption data for Pchlide (A-D), Y193F (E-H) and wild-type (I-L). The SADS (black dots) have been fitted with a sum (dark red line) of the following Gaussian functions of fixed position and FWHM (in brackets). Negative peaks: 1743 (30), 1687 (32), 1656 (32) (protein only) cm^{-1}. Positive peaks: 1640 (27), 1612 (36), 1578 (28), 1550 (34), 1521 (10), 1505 (20) cm^{-1}. Values of position and FWHM were derived from a fitting of free parameters to all datasets to find common values.

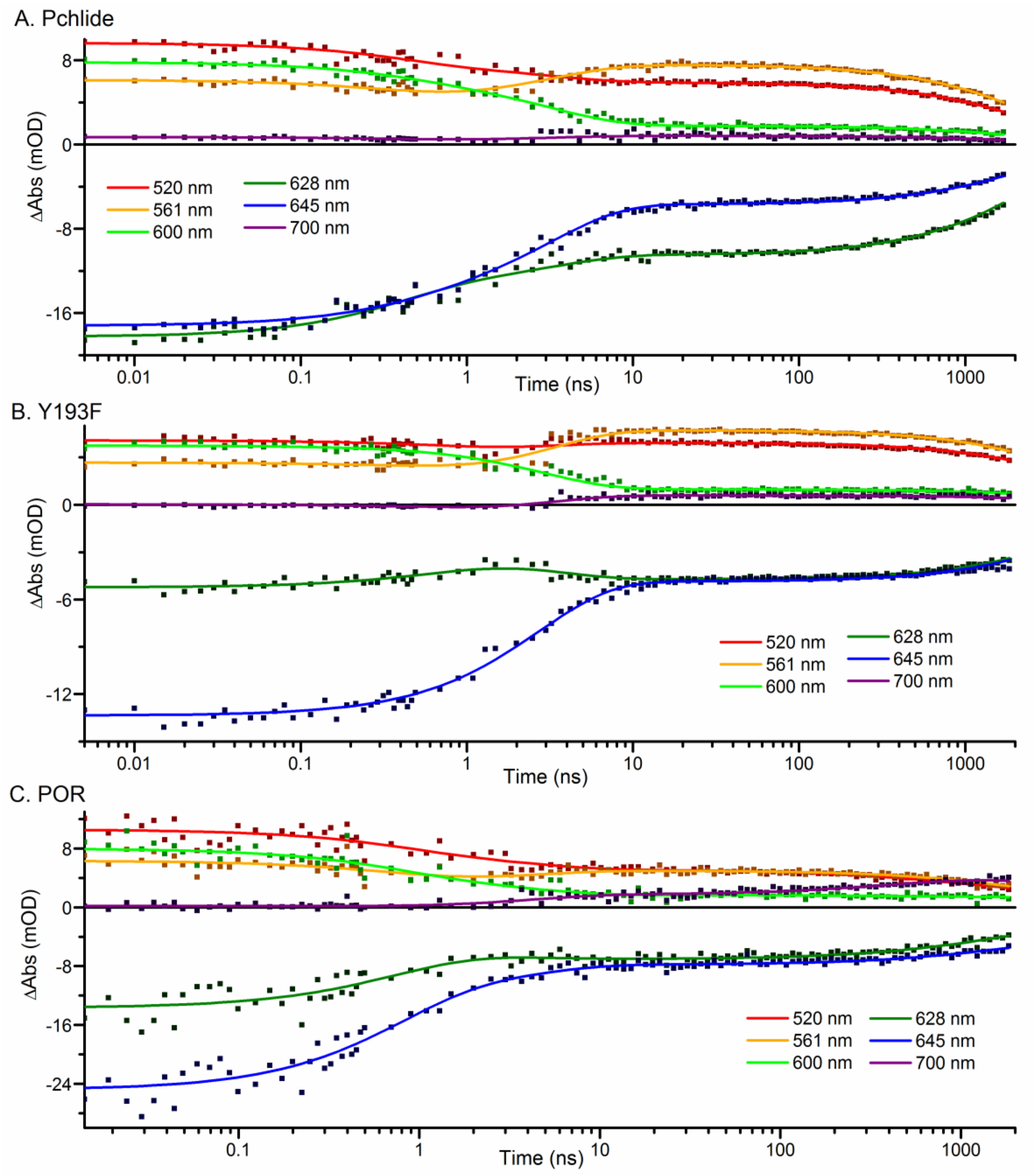

Figure S10. Kinetic traces at selected wavelengths with corresponding fits resulting from a global analysis of the time-resolved visible data for Pchlide only, theY193F-Pchlide-NADPH ternary complex, and the wild-type POR-Pchlide-NADPH ternary complex after excitation at 450 nm. The data were fitted as described in the Supporting Information.

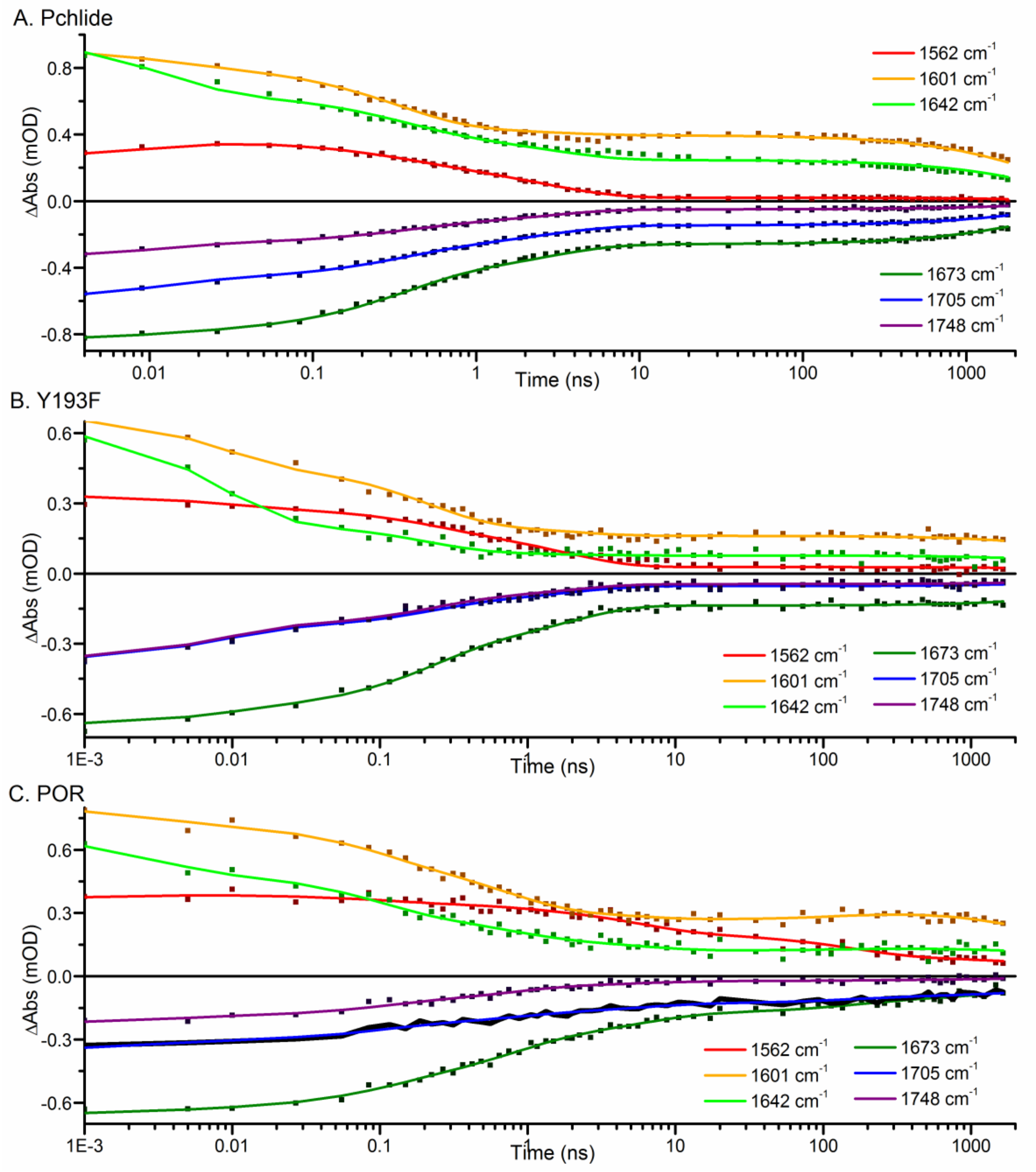

Figure S11. Kinetic traces at selected wavenumbers with corresponding fits resulting from a global analysis of the time-resolved IR data for Pchlide only, theY193F-Pchlide-NADPH ternary complex, and the wild-type POR-Pchlide-NADPH ternary complex after excitation at 450 nm. The data were fitted as described in the Supporting Information.

References:

1. Heyes, D. J. and Hunter, C. N. (2004) Identification and characterization of the product release steps within the catalytic cycle of protochlorophyllide oxidoreductase. *Biochemistry* 43:8265-8271.

2. Menon, B. R. K., Waltho, J. P., Scrutton, N. S., Heyes, D. J. (2009) Cryogenic and laser photoexcitation studies identify multiple roles for active site residues in the light-driven enzyme protochlorophyllide oxidoreductase. *J. Biol. Chem.* 284: 18160-18166.

3. Heyes, D. J., Hardman, S. J. O., Mansell, D., Gardiner, J. M., Scrutton, N. S. (2012) Mechanistic reappraisal of early stage photochemistry in the light-driven enzyme protochlorophyllide oxidoreductase. *PLoS One*, 7(9): e45642.

4. Greetham, G. M., Sole, D., Clark, I. P., Parker, A. W., Pollard, M. R., Towrie, M. (2012) Time-resolved multiple probe spectroscopy. *Rev. Sci. Instrum.*, *83*, 103107

5. Jones, A. R., Russell, H. J., Greetham, G. M., Towrie, M., Hay, S. and Scrutton, N. S. (2012) Ultrafast Infrared Spectral Fingerprints of Vitamin B-12 and Related Cobalamins. *J. Phys. Chem. A* 116:5586-5594.

6. Snellenburg, J. J., S. P. Laptenok, R. Seger, K. M. Mullen, and I. H. M. van Stokkum. (2012) Glotaran: A Java-Based Graphical User Interface for the R Package TIMP. *J. Stat. Softw.* 49: 1-22.

7. Hanf, R., Fey, S., Dietzek, B., Schmitt, M., Reinbothe, C., Reinbothe, S., Hermann, G., and Popp, J. (2011) Protein-Induced Excited-State Dynamics of Protochlorophyllide, *J. Phys. Chem. A 115*, 7873-7881.

8. Sytina, O. A., van Stokkum, I. H. M., Heyes, D. J., Hunter, C. N., van Grondelle, R., and Groot, M. L. (2010) Protochlorophyllide Excited-State Dynamics in Organic Solvents Studied by Time-Resolved Visible and Mid-Infrared Spectroscopy, *J. Phys. Chem. B 114*, 4335-4344.

9. Dietzek, B., Tschierlei, S., Hermann, G., Yartsev, A., Pascher, T., Sundstrom, V., Schmitt, M., and Popp, J. (2009) Protochlorophyllide a: A Comprehensive Photophysical Picture, *ChemPhysChem 10*, 144-150.
